# Evaluation of the Effects of Incorporating Long-Acting Subcutaneous Insulin Into the Standard Treatment Protocol for Diabetic Ketoacidosis in Children

**DOI:** 10.5812/ijem-139684

**Published:** 2024-03-11

**Authors:** Fatemeh Saffari, Ali Homaei, Venus Chegini, Amir Javadi, Victoria Chegini

**Affiliations:** 1 Department of Pediatrics, Children Growth Research Center, Research Institute for Prevention Non-communicable Diseases, School of Medicine, Qazvin University of Medical Sciences, Qazvin, Iran; 2 Beth Israel Deaconess Medical Center, Harvard Medical School, Harvard University, Boston, MA, US; 3 Department of Obstetrics and Gynecology, School of Medicine, Qazvin University of Medical Sciences, Qazvin, Iran; 4 Department of Community Medicine, Medical Microbiology Research Center, School of Medicine, Qazvin University of Medical Sciences, Qazvin, Iran

**Keywords:** Children, Diabetic Ketoacidosis, Diabetes Type 1, Pediatric Intensive Care Unit, Insulin Analogues, Basal Insulin, Clinical Trial

## Abstract

**Background:**

Despite the progress made in the treatment of type 1 diabetes, the incidence of diabetic ketoacidosis (DKA) in children is still increasing, and its management requires hospitalization in the pediatric intensive care unit (PICU). It is important to find a new and low-risk treatment method to shorten the recovery time from DKA.

**Objectives:**

This study aimed to evaluate the effectiveness and safety of integrating two different types of long-acting subcutaneous insulin into the standard treatment for DKA in children.

**Methods:**

The study was conducted in the PICU, and comprehensive monitoring was performed throughout the process. Patients aged between 2 and 15 years were divided into three groups: Two intervention groups receiving the addition of two types of long-acting insulin, Detemir and Glargine, to the standard treatment, and a control group. Each group consisted of 36 individuals. The impact of the intervention on the recovery time from DKA and the potential complications were investigated in all three groups.

**Results:**

The analysis of the results revealed a significant difference in the duration of exiting the acute phase among the groups. Additionally, the post-hoc test demonstrated that the recovery time for ketoacidosis in the Detemir arm was significantly shorter than in the standard arm (P = 0.008). However, it is important to note that there were no significant differences in the occurrence of common complications among the three study groups.

**Conclusions:**

Based on the findings, it appears that incorporating specific types of long-acting subcutaneous insulin into the standard treatment of DKA in children leads to a reduction in the resolution time of the acute phase of ketoacidosis. Importantly, this approach does not introduce additional complications. Consequently, it has the potential to optimize resource allocation and enhance patient care by freeing up beds in the PICU.

## 1. Background

Type 1 diabetes (T1DM) is one of the most common chronic diseases in childhood, and approximately 80,000 new cases occur annually in children and adolescents under 15 years of age in the world. The frequency of diabetic ketoacidosis (DKA) at the onset of T1DM has been reported from 12.8% to 80% worldwide (1, 2).

Diabetic ketoacidosis is a severe complication of T1DM in children, with mortality rates ranging from 0.3% to 0.5% in developed countries and even higher in less developed societies (approximately 10%) (3, 4). Despite advancements in insulin production and diabetes control, the hospitalization rate of diabetic patients with DKA continues to rise (5). Additionally, the COVID-19 pandemic has led to a significant increase in the hospitalizations of children with severe DKA (6).

In critical cases of DKA, admission to the intensive care unit (ICU), close monitoring, and continuous intravenous rapid-acting insulin infusion with appropriate dosing to achieve established goals are standard and safe practices endorsed by international consensus guidelines in the latest DKA management protocols (7, 8). However, a child's admission to a pediatric intensive care unit (PICU) can be highly stressful for both the child and their parents, and it also poses a significant financial burden for parents and insurance providers (9).

It appears that long-acting insulin analogs can be beneficial in aiding the transition from continuous intravenous (IV) insulin to subcutaneous (SC) maintenance therapy in individuals with DKA. Furthermore, children who receive insulin glargine alongside their treatment demonstrate a faster recovery from DKA, leading to shorter stays in the ICU and reduced treatment costs. These findings are supported by some studies (10, 11).

## 2. Objectives

This study investigated the impact of incorporating subcutaneous long-acting insulin into the standard treatment on the duration of DKA resolution, PICU stay, and potential intervention-related complications.

## 3. Methods

A prospective open-label randomized clinical trial introduction: This prospective study aimed to evaluate the impact of long-acting subcutaneous insulin on the revolution time and length of hospitalization in children with DKA. Additionally, the study aimed to assess the potential complications associated with this new intervention.

### 3.1. Study Design

An open-label randomized clinical trial was designed with three parallel arms. Patients were randomly assigned to one of three treatment groups using the random block sampling method.

### 3.2. Participants

Children diagnosed with DKA were included in the study.

### 3.3. Interventions

Group 1: Standard treatment alone (control group).

Group 2: Standard treatment plus one type of long-acting subcutaneous insulin.

Group 3: Standard treatment plus another type of long-acting subcutaneous insulin.

### 3.4. Outcome Measures

The primary outcomes of interest were the time of revolution from DKA and the length of ICU stay. Secondary outcomes included the incidence of complications associated with the new intervention.

This prospective study was designed as an open-label randomized clinical trial with three parallel arms. The primary objective of the study was to examine the impact of incorporating long-acting subcutaneous insulin into the standard treatment for children with DKA, with a focus on the duration of recovery and length of hospitalization. Additionally, the potential complications associated with this novel intervention were explored. To ensure unbiased allocation, the patients were grouped into one of three treatment arms using the random block sampling method.

### 3.5. Sample Size

The sample size for this study was calculated taking into consideration the primary endpoint, a type I error rate of 5%, and a type II error rate of 20%. The parameters and formula for calculating the sample size are as follows:

α = 0.05, 1-α = 0.95, β = 0.2, 1-β = 0.8, µ1 = 8.1, µ2 = 5.9.

The value of using parameters was extracted from the study by Sanoe Harrison et al. (12). Because three groups were examined in the present study, the sample size calculated by the above formula was adjusted and recalculated by the following formula. In the adjusted sample size formula, *n* is the sample size, which is calculated by comparing the mean of two groups, and *g* is the number of groups that are considered for comparison.

The required sample size in this study was determined at 30 patients in each study group. Considering a 15% attrition rate during the study, a total of 36 participants were included in each treatment group.

### 3.6. Population

The study population consisted of patients who were admitted to the academic Children's hospital of Qazvin University of Medical Sciences, Qazvin, Iran, and they were screened for the presence of acute DKA.

### 3.7. Diagnosis

Acute DKA was diagnosed based on the following criteria, and all three criteria were required to confirm the diagnosis (12):

• Hyperglycemia (blood glucose > 11 mmol/L [≈200 mg/dL])

• Venous pH < 7.3 or serum bicarbonate < 18 mmol/L

• Ketonemia (blood ß-hydroxybutyrate ≥ 3 mmol/L) or moderate or large ketonuria

### 3.8. Inclusion Criteria

Patients diagnosed with DKA were included in the study. Additional inclusion criteria encompassed an age range of 2 to 15 years, willingness to participate in the trial, and signed informed consent.

### 3.9. Exclusion Criteria

The following criteria led to the exclusion of patients from the study:

(1) Initiation of intravenous insulin in another hospital, (2) diagnosis of hyperosmolar non-ketotic hyperglycemia (HHNK), (3) previous or current treatment with systemic corticosteroids, (4) factors or underlying diseases predisposing to ketosis, such as metabolic disorders, growth hormone deficiency, adrenal insufficiency or ketotic hypoglycemia, and (5) children with evidence of infection in blood, urine, cerebrospinal fluid, throat, or tracheal aspiration cultures.

The study took place in the PICU of an academic-based children's hospital.

### 3.10. Intervention

The patients were randomly assigned to one of three treatment groups. In the standard treatment (standard arm), the patients received continuous regular insulin infusion at a rate of 0.05 - 0.1 units/kg/hour (13). In addition to the standard treatment, the second and third groups received a single dose of two different types of long-acting insulin analogs. The dose administered was 0.5 units per kilogram of body weight, administered subcutaneously less than 4 hours (as soon as possible) from the diagnosis of DKA. Additionally, if the ketoacidosis did not resolve within 24 hours, the same dose of the long-acting insulin analogs was repeated.

In the second group, the patients received insulin Detemir (Levemir® FlexPen®, Novo Nordisk Company, Denmark); however, the third group received insulin glargine (Lantus®, Sanofi Company, France).

The patients were then randomly assigned to one of the three treatment groups. Random assignment helps minimize bias and ensure that each participant has an equal chance of being assigned to any of the treatment groups. This randomization process helps improve the validity and reliability of the study's findings.

All patients were admitted to the PICU and underwent continuous pulmonary and cardiac monitoring throughout the treatment period to assess T-waves for any signs of hyper- or hypokalemia. Neurologic injury assessment was performed initially and at every hour of clinical signs of cerebral edema.

At the beginning of hospitalization, the degree of dehydration was evaluated according to clinical symptoms, and patients were divided into two groups with mild and moderate dehydration (5 - 10%) and severe dehydration (≥ 10%) (13). Blood glucose levels were measured and recorded every hour using a bedside glucometer. Additionally, blood samples were collected every 2 hours to analyze various parameters, such as serum or plasma glucose levels and electrolyte concentrations, and blood gas analysis, including pH, HCO_3_, and pCO_2_ (14).

For each patient in the three groups, we documented an hourly flow chart that included vital signs, clinical observations, the volume of fluids and intravenous drugs administered, laboratory results, duration of ketoacidosis resolution, length of stay in the PICU, occurrences of hypoglycemia, electrolyte disorders, clinical signs of cerebral edema, and the need for mannitol administration.

Hypoglycemia is defined as a glucose level of < 70 mg/dL (< 3.9 mmol/L), based on laboratory measurements or rapid capillary blood glucose obtained via glucometer, and is used as a threshold value to initiate hypoglycemia treatment (15). Hypokalemia was defined as a serum potassium concentration below 3.5 mmol/L (16).

Clinical diagnosis of cerebral injury was based on the examination and assessment of the neurological condition, and it was repeated at the patient's bedside. In the case of a diagnostic criterion, two main criteria or one main criterion and two minor criteria were given to diagnose cerebral edema, and mannitol was prescribed for the patient (13).

### 3.11. Study Outcomes

The study focused on two main outcomes. Firstly, the primary outcome was the average recovery time from the acute phase of ketoacidosis. To determine recovery, we assessed the achievement of venous pH ≥ 7.3, serum bicarbonate levels > 18 mmol/L, and, if applicable, successful dietary tolerance and absence of electrolyte disturbance. In cases where these criteria were met, we converted continuous intravenous insulin to subcutaneous insulin (12, 16). The duration of the ketoacidosis revolution was measured, from the initiation of continuous intravenous insulin until its discontinuation, in hours. The second outcome involved assessing and documenting any potential adverse events associated with the treatment protocols in each study arm. We also evaluated the necessity for additional treatment, if applicable.

### 3.12. Statistical Analysis

The Shapiro-Wilk test was used to assess the normality of quantitative data. Quantitative data were reported as mean (± standard deviation [SD]) or median (± interquartile range [IQR]); however, qualitative data were presented as percentages. A paired *t*-test was conducted to compare the mean difference of biochemical parameters before and after treatment within each arm. The chi-square test and Fisher's exact test were employed to examine the relationship between qualitative variables. The analysis of variance (ANOVA) was used to compare the mean time of recovery. A one-way ANCOVA was conducted to compare the effect of the three treatment regimens while controlling for the level of baseline biochemical parameters. Additionally, ANOVA was utilized to evaluate the trend of changes in the serum levels of blood factors. A significance level of 5% was chosen for all statistical analyses. Data analysis was conducted using SPSS software version 23 (SPSS Inc., Chicago, IL, USA).

### 3.13. Ethics

The study protocol was approved by the Institutional Ethics Committee of Qazvin University of Medical Sciences. Additionally, the study was registered in the Iranian Registry of Clinical Trials with the registration number IRCT20201125049485N1).

## 4. Results

In the present study, a total of 124 patients were screened, and 108 patients based on inclusion and exclusion criteria were recruited in the study. The patients were randomly allocated to one of the three treatment groups. All 36 patients in arm-1 (standard treatment), 36 patients in arm-2 (standard treatment + Detemir), and 36 patients in arm-3 (standard treatment + Glargine) successfully completed the study.

The demographic and baseline characteristics of each study arm are summarized in Table 1. The male-to-female ratio in the registered children was 52 males to 55 females. The average age of the participants was 8.3 ± 3.1 years within a range of 2 - 14 years. On average, the patients weighed 27.5 ± 10.9 kg (range: 13 - 51 kg) and had an average height of 124.7 ± 19.1 cm (range: 91 - 158 cm). Most patients did not have a family history of T1DM. In terms of the pubertal stage, 91 (85.1%), 10 (9.4%), and 6 (5.5%) patients were in the first stage, the second stage, and the third or higher stages, respectively. Additionally, Table 1 shows the baseline blood biochemical and clinical parameters, which include blood glucose, sodium, potassium, blood urea nitrogen (BUN), creatinine, white blood cell (WBC), neutrophils, GCS, systolic blood pressure, anion gap, ketonuria, and hydration. There were no statistically significant differences in demographic and baseline characteristics among patients randomly assigned to each treatment arm (P > 0.05).

**Table 1.** Demographic and Baseline Characteristics of Subjects for Each Arm ^a^

Variables	Standard (n = 36)	Detemir (n = 36)	Glargine (n =36)	P-Value
**Gender**				0.337
Male	21 (58.3)	15 (41.7)	16 (45.7)	
Female	15 (41.7)	21 (58.3)	19 (54.3)	
**Age (y)**	8.0 ± 3.2	8.5 ± 3.2	8.3 ± 3.0	0.795
**Weight (kg)**	27.4 ± 11.1	28.61 ± 11.9	26.5 ± 9.5	0.724
**Height (cm)**	123.7 ± 19.7	126.9 ± 19.9	123.3 ± 18.3	0.679
**BMI (kg/m** ^**2**^ **)**	17.1 ± 2.6	17.2 ± 3.9	17.0 ± 1.9	0.935
**History of family diabetes **				0.837
No	30 (83.3)	28 (77.8)	28 (80.0)	
Yes	6 (16.7)	8 (22.2)	7 (20.0)	
**Puberty stage ** ^**b**^				0.798
I	31 (86.1)	30 (83.3)	30 (85.7)	
II	2 (5.6)	4 (11.1)	4 (11.4)	
III	2 (5.6)	2 (5.6)	1 (2.9)	
IV	1 (2.8)	0 (0.0)	0 (0.0)	
**Blood sugar (mg/dL) ** ^**c**^	485.5 ± 134.6 (290 - 796)	538.0 ± 129.7 (284 - 846)	478.5 ± 134.9 (297 - 781)	0.123
**Sodium (mmol/L) ** ^**c**^	132.8 ± 3.5 (126 - 141)	131.6 ± 3.4 (125 - 141)	133.4 ± 3.3 (124 - 140)	0.075
**Potassium (mmol/L)** ^**c**^	4.4 ± 0.7 (3.0 - 5.6)	4.4 ± 0.6 (3.2 - 5.8)	4.2 ± 0.6 (3.3 - 5.5)	0.285
**BUN (mg/dL) ** ^**c**^	18.9 ± 4.3 (8-26)	18.2 ± 5.1 (9-29)	19.5 ± 4.8 (9-29)	0.481
**Creatinine (mg/dL) ** ^**c**^	1.2 ± 0.3 (0.8 - 2.2)	1.3 ± 0.3 (0.7 - 2.2)	1.3 ± 0.4 (0.7 - 2.1)	0.828
**WBC ** ^**d**^	16300 (13400 - 18950)	14700 (12150 - 20600)	14950 (11600 - 16300)	0.201
**Neutrophils**	73.4 ± 13.7	72.4 ± 11.7	74.3± 11.9	0.808
**GCS ** ^**b**^	13.6 ± 1.2 (11 - 15)	13.9 ± 1.1 (10 - 15)	13.5 ± 1.2 (11 - 15)	0.312
**Anion gap (mmol/L)**	23.9 ± 2.5 (18 - 28)	23.9 ± 2.8 (19 - 28)	24.1 ± 2.5 (19 - 28)	0.913
**Ketonuria**				0.999
Moderate	13 (36.1)	13 (31.6)	12 (33.3)	
Severe	23 (63.9)	23 (63.9)	24 (66.7)	
**Dehydration degree**				0.939
Severe	14 (38.9)	14 (38.9)	15 (41.6)	
Mild to moderate	22 (61.1)	22 (61.1)	21 (58.4)	
**Systolic blood pressure ** ^**b**^ ** (percentile for height)**				0.978
25th – 75th	15 (41.7)	16 (44.4)	17 (47.2)	
75th – 90th	16 (44.4)	16 (44.4)	16 (44.5)	
90th – 95th	5 (13.9)	4 (11.1)	3 (8.3 )	

Abbreviations: BMI, body mass index; BUN, blood urea nitrogen; WBC, white blood cell.

^a^ Values are presented as No. (%) or mean ± SD.

^b^ Fisher’s exact test.

^c^ Baseline blood biochemistry parameter values.

^d^ Median (quartile 1-quartile 3).

To assess the impact of the three treatment regimens while accounting for the baseline levels of biochemical parameters, a one-way ANCOVA was performed on the average revolution time of the estimated biochemical parameters. The results indicated that there was no significant difference between the intervention groups in terms of the average baseline biochemical parameters.

Despite no significant differences in the mean venous blood gas (VBG) measurements, including pH and HCO_3_, at the time of DKA diagnosis and other biochemical parameters at the time of recovery between the groups, the analysis results revealed a significant disparity in the mean recovery time among the treatment groups (P = 0.008). Specifically, the post-hoc test indicated that the Detemir arm exhibited a significantly shorter recovery time from DKA than the standard arm (P = 0.011), as demonstrated in Table 2. 

**Table 2.** Comparison of Outcome Stratified by Treatment Group

Variables ^a^	Standard	Detemir	Glargine	P-Value
**Blood sugar (mg/dL)**	193.3 ± 39.4 (136 - 293)	179.3 ± 33.4 (103 - 245)	181.1 ± 36.8 (122 - 264)	0.218
**Sodium (mmol/L)**	141.4 ± 2.6 (136 - 146)	140.5 ± 3.0 (134 - 145)	140.6 ± 2.9 (136 - 146)	0.344
**Potassium (mmol/L)**	3.9 ± 0.5 (3 - 4.9)	3.9 ± 0.5 (3.1 - 5.3)	4.0 ± 0.5 (3.1 - 5.2)	0.556
**BUN (mg/dL)**	14.5 ± 3.3 (9 - 24)	14.2 ± 3.7 (8 - 22)	14.0 ± 3.3 (9 - 22)	0.141
**Creatinine (mg/dL)**	0.8 ± 0.2 (0.6 - 1.2)	0.8 ± 0.2 (0.4 - 1.2)	0.9 ± 0.2 (0.5 - 1.3)	0.085
**DKA duration (hour)**	27.4 ± 12.2 (10 - 50)	19.5 ± 9.7 (6 - 43)	24.1 ± 10.6 (7 - 42)	0.011
**VBG pH**	7.1 ± 0.1 (6.9 - 7.4)	7.2 ± 0.1 (6.9 - 7.3)	7.2 ± 0.1 (6.9 - 7.3)	0.242
**VBG_HCO**_**3**_ ** (mmol/L)**	7.9 ± 3.5 (2.8 - 14.2)	8.3 ± 3.2 (3.2 - 14.5)	8.4 ± 3.7 (2.9 - 14.5)	0.794

Abbreviations: BUN, blood urea nitrogen; DKA, diabetic ketoacidosis; VBG, venous blood gas.

^a^ Variables, including blood sugar, sodium, potassium, BUN, and creatinine, were at the time of the DKA revolution. VBG parameters, including pH and HCO_3_ at the time of diagnosis of DKA and initiation of treatment.

Although the analysis revealed a reduction in recovery time, no significant correlation was observed between each treatment arm and the occurrence of side effects. These side effects included the frequency of cerebral edema (requiring mannitol), hypoglycemia, and hypokalemia during the treatment (Table 3). 

**Table 3.** Side Effects of Treatment Stratified by Treatment Group ^a^

Variables	Standard (n = 36)	Detemir (n = 36)	Glargine (n = 36)	P-Value
**Mannitol**				0.852
No	27 (75.0)	29 (80.6)	28 (77.8)	
Yes	9 (25.0)	7 (19.4)	8 (22.2)	
**Hypoglycemia ** ^**b**^				0.458
No	33 (91.7)	32 (88.9)	29 (80.6)	
Yes	3 (8.3)	4 (11.1)	7 (19.4)	
**Hypokalemia (baseline) ** ^**b**^				0.695
Yes	3 (8.3)	1 (2.8)	3 (8.3)	
No	33 (91.7)	35 (97.2)	33 (91.7)	
**Hypokalemia (after treatment)**				0.954
Yes	7 (19.4)	8 (22.2)	7 (19.5)	
No	29 (80.6)	28 (77.8)	29 (80.5)	

^a^ Values are presented as No. (%).

^b^ Fisher’s exact test.

## 5. Discussion

With the growing prevalence of diabetes cases among children and the subsequent rise in hospital admissions due to DKA (5, 6), it is crucial to explore solutions that promote faster recovery without adverse effects. Such interventions have the potential to reduce costs and shorten the duration of ICU stays. Additionally, they can greatly alleviate emotional stress for both parents and children involved.

Based on the importance of the subject, a decision has been made to further investigate the effectiveness and safety of including long-acting subcutaneous insulin in the standard treatment for expediting the recovery process of DKA in children. This research aimed to obtain valuable insights into the potential benefits and possible side effects of using insulin detemir and insulin glargine in this particular context.

In this study, it was observed that the concurrent administration of basal insulin with continuous infusion of regular insulin was well tolerated and linked to a quicker recovery from DKA without any complications. The data indicated that the group receiving simultaneous administration of insulin Detemir experienced a shorter duration for resolving ketoacidosis. Although the incidence of severe hypoglycemia was not statistically significant in the insulin glargine group, the faster reduction in blood sugar levels resulted in a decrease in the continuous short-acting insulin dose. Consequently, this led to an increase in the recovery time from ketoacidosis.

Limited research has been conducted on the inclusion of subcutaneous long-acting insulin in the standard treatment protocol for DKA, which typically involves continuous infusion of short-acting insulin. Additionally, to the best of our knowledge, no study has been conducted using the present specific methods in children, and the comparison of the effects of different types of long-acting insulin has not been explored in any of these studies. Two articles with similar findings have focused on children within a specific age range (12, 17); however, other studies have been conducted in the adolescent or adult age group (18-21).

In a study involving 129 diabetic patients, the impact of incorporating long-acting insulin (glargine) into the standard treatment for DKA was examined. The study concluded that there were similar improvements in acidosis and incidence of hypoglycemia between the groups. However, the group receiving additional glargine insulin had a higher incidence of hypokalemia than the standard group. It is worth noting that cerebral edema occurred in 3.6% of the patients in the standard group; nevertheless, none of the patients receiving glargine experienced this complication (12).

The current study revealed that incorporating long-acting insulin into the standard treatment protocol for DKA did not lead to an increase in side effects, such as cerebral edema, hypokalemia, or severe hypoglycemia. Additionally, the present study showed a significant reduction in the recovery time from the acute phase when regular insulin and insulin detemir were administered concurrently. In a small-scale study involving children with DKA, acidosis resolved at a faster rate without any negative consequences in those who received an additional 0.3 units per kilogram of subcutaneous insulin (glargine) alongside standard therapy (17).

In a randomized controlled trial involving adults, the addition of insulin glargine to standard DKA therapy showed a reduction in the average time to recovery from DKA without experiencing hypoglycemic and hypokalemic episodes. However, this reduction in time was not statistically significant, likely due to the small sample size (18). Another prospective randomized clinical trial in adults demonstrated that subcutaneous administration of insulin glargine within the first 12 hours of intravenous insulin infusion significantly decreased rebound hyperglycemia after discontinuation of intravenous insulin, with no adverse effects. It is important to note that the patients included in this study were not necessarily in the ketoacidosis stage, and therefore, the impact of adding subcutaneous insulin to intravenous infusion on shortening the DKA phase remains undetermined (19).

In a separate study examining the impact of incorporating glargine insulin into the standard treatment for DKA, it was observed that the average recovery time from the DKA phase was 10.2 hours in the glargine group, compared to 11.6 hours in the control group. Additionally, the estimated average length of hospitalization was 3.9 and 4.6 days in the glargine and control groups, respectively. However, the rates of hospitalization in the ICU and occurrences of hypoglycemia were similar between the two groups. This information is supported by another study (20). In a similar study conducted on adults with DKA associated with type 2 diabetes, it was observed that the inclusion of insulin glargine within the first 3 hours of standard treatment resulted in a reduction in the recovery time and the occurrence of rebound hyperglycemia. Importantly, this intervention did not lead to an increase in side effects. This information is supported by another study (21).

Based on the findings of the present study, it is suggested that the inclusion of detemir insulin in the standard treatment has the potential to decrease the length of hospitalization in the ICU and accelerate the recovery time of DKA without an increase in complications. The aforementioned results indicate that utilizing detemir insulin treatment might be more efficacious in reducing the recovery time than the standard treatment, with minimal adverse effects.

Recognizing the limitations is crucial when interpreting the results of the study. One of the limitations mentioned is the failure to categorize DKA patients into different groups based on the severity of acidosis, which could have influenced the outcomes. It is important to consider these limitations when evaluating the findings of the study. Furthermore, conducting additional research with a larger sample size while taking these limitations into account would provide more robust evidence to support the conclusions drawn. This issue highlights the need for further research in this field to gain a better understanding of the potential benefits and drawbacks of incorporating long-acting insulin into the treatment plan for DKA, particularly in pediatric patients.

### 5.1. Conclusions

In conclusion, the present study suggests that adding long-acting subcutaneous insulin to children with DKA might positively affect their recovery time and length of hospitalization. However, further research is necessary to assess potential complications associated with this new intervention.

## Data Availability

The dataset presented in the study is available on request from the corresponding author during submission or after publication.

## Clinical Trial Registration Code:

(IRCT20201125049485N1).
